# BARRIERS TO SUCCESSFUL AGING AMONG OLDER WOMEN AND MEN LIVING WITH HIV

**DOI:** 10.1093/geroni/igad104.1347

**Published:** 2023-12-21

**Authors:** Anna Rubtsova, Tonya Taylor, Gina Wingood, Ighovwerha Ofotokun, Deborah Gustafson, David Vance, Dilip Jeste, David Moore

**Affiliations:** Emory University, Rollins School of Public Health, Atlanta, Georgia, United States; SUNY Downstate Medical Center, Brooklyn, New York, United States; Columbia University, New York City, New York, United States; Emory University School of Medicine, Atlanta, Georgia, United States; SUNY Downstate, Brooklyn, New York, United States; University of Alabama at Birmingham, Birmingham, Alabama, United States; University of California, San Diego, San Diego, California, United States; University of California at San Diego, San Diego, California, United States

## Abstract

This study identified and compared barriers to successful aging (SA) among older women and men living with HIV. Participants were recruited through 1) the Women’s Interagency HIV Study (WIHS), Atlanta and Brooklyn sites; and 2) the HIV Neurobehavioral Research Program at the University of California, San Diego. Our sample included 31 participants living with HIV: 17 women and 14 men, ages 52 to 73 years, 58% African American. We conducted semi-structured interviews, exploring SA with HIV. The interviews (~90 minutes long) were audio recorded, fully transcribed, and imported into MAXQDA software for analysis. Thematic and comparative analyses were conducted within a social-constructivist framework. We uncovered several themes related to SA barriers that were shared by women and men: challenging chronic conditions, stigmas, substance use, and lack of resources. We also found differences. While men stressed dreading old age and loneliness, women emphasized stress and ‘giving up’ as barriers to SA.

